# Understanding Transitions in IADL and Their Consequences for Cognitive Impairment Among Older Mexican Americans

**DOI:** 10.1093/geroni/igaa057.1070

**Published:** 2020-12-16

**Authors:** Jiwon Kim, Jacqueline Angel, Sunshine Rote

**Affiliations:** 1 University of Texas, AUSTIN, Texas, United States; 2 The University of Texas at Austin, Austin, Texas, United States; 3 University of Louisville, Louisville, Kentucky, United States

## Abstract

Mexican Americans tend to live longer lives than other ethnic groups, but it remains unclear how this trend influences the trajectory of disability and its consequences for cognitive frailty. Building on previous research, we assess transitions in IADL among the oldest old. We use data from three waves of the Hispanic Established Population of the Epidemiologic Study of the Elderly (H-EPESE) to investigate trajectories of IADL disability as individuals’ age into their 80s and 90s, a period of the life course with much higher rates of morbidity and decreasing socioeconomic resources. The H-EPESE is a benchmark longitudinal cohort study based on an original sample of 3,050 Mexican-Americans aged 65 and older in the Southwestern United States. Our modeling approach estimates transitions in patterns of IADL employing the Latent Transition Analysis (LTA). Results revealed three heterogeneous latent classes: high IADLs, difficulty in transportation and mobility, and low IADLs. Those with high IADLs tended to remain in the same class over time. Individuals having difficulty in transportation and mobility tended to stay in the same class or transfer to high IADLs, whereas those with overall low IADLs transferred to either the same class or to difficulty in transportation and mobility. Additional analysis revealed that cognitive impairment was a significant predictor of instrumental disability over time. Furthermore, females were more likely than males to belong to difficulty in transportation or mobility class than to the low IADL class. Our results highlight the long term consequences of cognitive decline on IADL limitations.

